# Circulating microRNAs and endothelial cell migration rate are associated with metabolic syndrome and fitness level in postmenopausal African American women

**DOI:** 10.14814/phy2.14173

**Published:** 2019-07-25

**Authors:** Ryan M. Sapp, Daniel D. Shill, Chiranjeev Dash, Jennifer C. Hicks, Lucile L. Adams‐Campbell, James M. Hagberg

**Affiliations:** ^1^ Department of Kinesiology, School of Public Health University of Maryland College Park Maryland; ^2^ Georgetown Lombardi Comprehensive Cancer Center, Office of Minority Health & Health Disparities Research Washington District of Columbia

**Keywords:** Cardiorespiratory fitness, circulating microRNA, endothelium, menopause, metabolic syndrome

## Abstract

Postmenopausal African American women are at elevated risk for metabolic syndrome (MetS), which predisposes them to cardiovascular disease and other chronic diseases. Circulating microRNAs (ci‐miR) are potential mediators of cardiometabolic diseases also impacted by cardiorespiratory fitness (CRF) level. Using real‐time quantitative PCR, we compared the expression of vascular‐related ci‐miRs (miR‐21‐5p, miR‐92a‐3p, miR‐126‐5p, miR‐146a‐5p, miR‐150‐5p, miR‐221‐3p) in sedentary, overweight/obese, postmenopausal African American women based on 1) presence (*n* = 31) or absence (*n* = 42) of MetS and 2) CRF level (VO_2peak_) (Very Low < 18.0 mL·kg^−1^·min^−1^ [*n* = 31], Low = 18.0–22.0 mL·kg^−1^·min^−1^ [*n* = 24], or Moderate >22.0 mL·kg^−1^·min^−1^ [*n* = 18]). Endothelial migration rate in response to subjects’ serum was assessed to determine the effect of circulating blood‐borne factors on endothelial repair. Ci‐miR‐21‐5p was the only ci‐miR that differed between women with MetS compared to those without MetS (0.93 ± 0.43 vs. 1.28 ± 0.71, *P* = 0.03). There were borderline significant differences (*P* = 0.06–0.09) in ci‐miR‐21‐5p, 126‐5p, and 221‐3p levels between the CRF groups, and these three ci‐miRs correlated with VO_2peak_ (*r* = −0.25 to −0.28, *P* < 0.05). Endothelial migration rate was impaired in response to serum from women with MetS compared to those without after 16–24 h. Serum from women with Moderate CRF induced greater endothelial migration than the Very Low and Low CRF groups after 4 and 16–24 h, that was also not different from a young, healthy reference group. Ci‐miR‐21‐5p is lower in postmenopausal African American women with MetS, while ci‐miRs‐21‐5p, 126‐5p, and 221‐3p are associated with CRF. Factors which impair endothelial cell migration rate are present in serum of women with MetS, though having Moderate CRF may be protective.

## Introduction

The menopausal transition is associated with an increase in prevalence of the metabolic syndrome (MetS) (Janssen et al. 2008), a clustering of interrelated risk factors which doubles the risk for cardiovascular disease (CVD) (Mottillo et al. 2010). While about one‐third of postmenopausal women have MetS (Hallajzadeh et al. 2018), African American women, specifically, exhibit greater severity of MetS (Gurka et al. 2016) and higher rates of CVD (Carnethon et al. 2017) as compared to their Caucasian counterparts. Major underlying risk factors for MetS include abdominal obesity, insulin resistance, and physical inactivity, all of which are more prevalent in African Americans (Grundy et al. 2005; Khan et al. 2015; Malayala and Raza, 2016). According to NHANES data, more than two‐thirds of African American women present abdominal obesity (Moore et al. 2017). Other risk factors, including aging and hormonal imbalance, put postmenopausal African American women at very high risk for MetS (Grundy et al. 2005; Mesch et al. 2006; Clark and El‐Atat, 2007). Furthermore, while high cardiorespiratory fitness (CRF) levels are protective against CVD and mortality, African American women on average are less fit than Caucasian women (Skinner et al. 2001; LaMonte et al. 2002; Ribisl et al. 2007; Swift et al. 2013). Lower CRF levels may contribute to the more rapid progression and greater severity of MetS (Gurka et al. 2016), as well as the higher CVD prevalence and associated mortality that are apparent in African American as compared to Caucasian women (Carnethon et al. 2017), although specific mechanisms remain to be fully elucidated.

The blood‐borne circulating milieu is unfavorably altered with MetS, including factors that contribute negatively to vascular health (Briançon‐Marjollet et al. 2014; Chedraui et al. 2014; Ahirwar et al. 2015). One such recently recognized class of circulating potential factors are microRNAs (miRs) – short, non‐coding RNAs that regulate gene expression at the post‐transcriptional level via complimentary binding to target mRNAs. Several miRs play key roles in the cardiovascular system, especially the endothelium, where they regulate function and the atherogenic process (Wu et al. 2009; Nishiguchi et al. 2015; Araldi and Suárez, 2016). MiRs can be released by cells into the bloodstream as circulating (ci)‐miRs and can be subsequently taken up by the endothelium to impact these processes (Zernecke et al. 2009; Zhang et al. 2010; Jansen et al. 2013; Dlouhá and Hubáček, 2017; Njock and Fish, 2017; Bär et al. 2019). There is a large body of research showing that ci‐miRs may act as biomarkers and intercellular mediators of cardiometabolic disorders (Dlouhá and Hubáček, 2017; Njock and Fish, 2017; Bär et al. 2019). Previous studies also suggest that certain ci‐miRs are associated with CRF (Sapp et al. 2017; Sapp and Hagberg, 2019). Thus, ci‐miRs altered with MetS and CRF may partially mediate the development of cardiovascular disease.

The expression levels of vascular‐related ci‐miRs in postmenopausal African American women, and their associations with MetS and CRF are currently unexplored. Therefore, the purpose of this study was to determine associations of MetS and CRF with ci‐miRs related to vascular health in overweight/obese, postmenopausal African American women. We further sought to explore the effects of the total blood‐borne circulating factors in these subjects on endothelial cell repair function by assessing endothelial cell migration in response to subjects’ serum. We hypothesized that serum from women without MetS and with higher CRF would induce faster and more complete migration than serum of women who had MetS and were less fit.

## Methods

### Subjects

Information on participant recruitment has been previously described in detail (Adams‐Campbell et al. 2016). The study was initially approved by the institutional review board (IRB) at Georgetown University. The use of stored serum samples for experiments was then approved by the University of Maryland IRB. Informed consent was obtained from all individuals before participation. Participants were African American women between the ages of 40–65 who were postmenopausal (last menstrual period ≥12 months), sedentary (<60 min of physical activity a week for the past 6 months), and overweight or obese (BMI ≥ 25 kg m^−^^2^). Participants reported that they were not currently using exogenous female hormones. Blood was drawn in the morning after an overnight fast. Blood samples were spun at 3000 rpm for 10 min at 4°C and serum was stored in aliquots at −80°C. The volume of serum samples was limiting for some subjects, so although our entire sample pool included 73 subjects, some samples used for ci‐miR analysis were not used for endothelial migration experiments and vice‐versa. We used samples from 62 subjects for each assay, obtaining both ci‐miR and migration data for 51 of the subjects. The number of subjects in each group was kept similar for each assay.

For endothelial migration experiments, a healthy group of premenopausal, physically inactive to moderately active women were recruited and tested similarly, in order to determine a reference, “near‐optimal” level for endothelial cell migration rate. All young women were devoid of MetS components and CVD risk factors.

### Metabolic syndrome assessment

The presence of MetS was determined based on the National Cholesterol Education Program’s Adult Treatment Panel III revised guidelines (Grundy et al. 2005). Women having at least three of the following were diagnosed with MetS: waist circumference >88 cm, serum triglyceride ≥150 mg dL^−^^1^, HDL‐C <50 mg dL^−^^1^, blood pressure ≥130/≥85 mmHg, serum glucose ≥100 mg dL^−^^1^. Specific information on testing for individual components has been published previously (Adams‐Campbell et al. 2016).

### Cardiorespiratory fitness tests

In order to assess CRF, participants completed a maximal oxygen consumption (VO_2max_) test using the Bruce treadmill protocol. Following a 5‐minute warmup, speed and grade were increased every 3 min until volitional exhaustion. Throughout the exercise period, expired air was analyzed using a ParvoMedics TrueOne 2400 metabolic cart (Sandy, UT) and heart rate was tracked with a POLAR H7 Bluetooth Smart Heart Rate monitor (Gays Mills, WI). The majority of subjects did not meet the criteria for a true VO_2max_ (plateau in VO_2_, respiratory exchange ratio (RER) >1.15, and age‐predicted maximal heart rate) and therefore values are reported as VO_2peak_. Participants were categorized into tertiles based on VO_2peak_, defined as having Very Low (<18.0 mL·kg^−1^·min^−1^), Low (=18.0–22.0 mL·kg^−1^·min^−1^), or Moderate (>22.0 mL·kg^−1^·min^−1^) CRF (Adams‐Campbell et al. 2016).

### Circulating microRNA quantification

Serum samples were thawed at room temperature and centrifuged at 16,000*g* for 10 min at 4°C to remove cell debris. Total RNA was isolated from 200 µL serum using the miRNeasy serum/plasma kit according to the manufacturer’s protocol (Qiagen, Germantown, MD). A spike‐in control miR (C. elegans miR‐39) was added to all serum samples during isolation for assessment of recovery and calibration of PCR results. Reverse transcription was performed (miScript II RT kit, Qiagen) using 4 µL RNA and the final reaction mix was diluted in 200 µL RNase‐free water. Real‐time quantitative PCR was run on an ABI 7300 Real‐Time PCR System (Applied Biosystems) using 2.5 µL input cDNA with the miScript SYBR Green PCR Kit (Qiagen, Germantown, MD). The expression levels of six ci‐miRs (21‐5p, 92a‐3p, 126‐5p, 146a‐5p, 150‐5p, 221‐3p), chosen *a priori* based on known associations with MetS, CRF, endothelial cell function, and/or inflammation (Bye et al. 2013; Zhou et al. 2014; Nishiguchi et al. 2015; Araldi and Suárez, 2016; He et al. 2016; Dlouhá and Hubáček, 2017; Njock and Fish, 2017; Sapp et al. 2017), were assessed using specific Qiagen miScript primer assays (MS00009079, MS00006594, MS00006636, MS00003535, MS00003577, MS00003857). Groups were compared using the 2^‐ΔΔCT^ method of relative quantification. For each miR, ΔCT = CT of miR – CT of spike‐in control miR; ΔΔCT = ΔCT for individual subject – average ΔCT of Moderate CRF group. All miRs were sufficiently detected at CT <35 in all samples.

### Endothelial cell migration assay

Human umbilical vein endothelial cells (HUVECs) from pooled donors (Lonza) were recovered from cryopreservation and passed once before use in experiments. Serum from individual subjects was applied to HUVECs (passages 3–5) in a gap closure assay (Radius 96‐Well Cell Migration Assay, Cell Biolabs, Inc., San Diego, CA) at a 5% concentration in endothelial growth medium (EGM‐2, Lonza) that did not contain fetal bovine serum (FBS). The percentage of human serum used was based on dose‐response experiments (data not shown). Subjects’ serum was assessed in duplicate. Growth media devoid of serum was used as a negative control condition. Migration was tracked by taking pictures under a light microscope every 4 h over 24 h, area measured using ImageJ (NIH), and reported as percentage of initial gap area covered (100 – (size of wound area at time point/size of initial wound area × 100)).

### Statistics

One‐way ANOVAs followed by pre‐planned contrasts were used to compare subject characteristics and ci‐miR expression between CRF groups. Characteristics of subjects grouped based on MetS were compared using independent *t*‐tests. Pearson’s correlation coefficients were calculated to assess correlations. Two‐way repeated measures ANOVAs (time × group) followed by preplanned contrasts were used to determine differences in endothelial migration. Area under the curve (AUC) was calculated for endothelial migration using GraphPad Prism 8. One‐way ANOVAs with pre‐planned contrasts were used to compare AUC between groups. The no serum condition was not included in any ANOVAs. All tests were two‐sided. Statistical significance was set at *P* < 0.05, while *P*‐values >0.05, but <0.10, were regarded as approaching statistical significance.

## Results

### Subject characteristics

Comparisons between groups based on presence of MetS (Table 1) revealed no significant differences in VO_2peak_ nor percent body fat, though the group with MetS had significantly lower (*P* = 0.04) VO_2peak_ relative to FFM than the Non‐MetS group. Women without MetS had lower systolic and diastolic blood pressure, fasting glucose and triglycerides, as well as higher HDL than those women with MetS (all *P* < 0.05).

**Table 1:** Characteristics of subjects grouped based on presence of Metabolic Syndrome (MetS).

	MetS (*n* = 31)	Non‐MetS (*n* = 42)
Age, y	50.6 ± 3.9	50.2 ± 4.4
VO_2peak_, L·min^−1^	1.9 ± 0.4	1.9 ± 0.4
VO_2peak_, mL·kg^−1^·min^−1^	18.8 ± 3.9	20.0 ± 5.2
VO_2peak_, mL·kg FFM^−1^·min^−1^	35.3 ± 6.9	39.1 ± 8.0*
Weight, kg	101 ± 20.8	99.2 ± 22.3
BMI, kg m^−2^	37.1 ± 7.0	36.4 ± 7.8
Body fat %	46.1 ± 5.5	47.6 ± 6.0
Waist Circumference, cm	113.6 ± 16.1	111.6 ± 17.1
Total FFM, kg	52.8 ± 8.4	50.0 ± 8.2
Leg FFM, kg	17.2 ± 2.8	16.1 ± 2.7
SBP, mmHg	128.1 ± 17.0	119.1 ± 13.9*
DBP, mmHg	85.1 ± 9.1	77.1 ± 8.1*
Glucose, mg dL^−1^	112.2 ± 26.4	93.2 ± 10.4*
Total cholesterol, mg dL^−1^	182.3 ± 52.8	193.3 ± 40.9
HDL‐C, mg dL^−1^	46.8 ± 12.1	61.1 ± 13.5*
LDL‐C, mg dL^−1^	109.3 ± 43.9	116.4 ± 41.5
Triglycerides, mg dL^−1^	131.0 ± 54.0	78.6 ± 36.4*

Means ± SD. *P < 0.05.

Characteristics of subjects grouped by CRF are shown in Table 2. There was approximately a 5 mL·kg^−1^·min^−1^ average difference in relative VO_2peak_ between adjacent CRF levels (*P* < 0.001). The significant differences observed in relative VO_2peak_ between the Moderate and Low CRF groups were lost when body weight was not taken into account (absolute VO_2peak_ [L·min^‐1^]). When VO_2peak_ was adjusted for FFM however, stepwise differences between groups remained (*P* < 0.001). Importantly, there were no significant differences between the Moderate, Low, or Very Low CRF groups in any criteria used to assess exertion in the VO_2peak_ test, including maximal heart rate (HR_max_) (162 ± 15 vs. 158 ± 20 vs. 156 ± 21 bpm), respiratory exchange ratio (RER_max_) (1.04 ± 0.11 vs. 1.03 ± 0.09 vs. 0.97 ± 0.13 VCO_2_∙VO_2_^−^^1^), or rating of perceived exertion (RPE_max_) (14.1 ± 1.6 vs. 14.4 ± 1.9 vs. 13.8 ± 1.8). Women with Moderate CRF levels had lower weight, BMI, body fat, and waist circumference than women in the other two groups (all *P* < 0.05), while women with Low CRF also had lower BMI and body fat than women with Very Low CRF (*P* < 0.05).

**Table 2:** Characteristics of subjects grouped by cardiorespiratory fitness (CRF).

	Very low CRF <18 mL·kg^−1^·min^−1^ (*n* = 31)	Low CRF 18–22 mL·kg^−1^·min^−1^ (*n* = 24)	Moderate CRF >22 mL·kg^−1^·min^−1^ (*n* = 18)	Young healthy (*n* = 7)
Age, year	49.8 ± 4.0	50.7 ± 4.6	50.9 ± 4.2	27.9 ± 4.3
VO_2peak_, L·min^−1^	1.7 ± 0.4	2.0 ± 0.4^*^	2.1 ± 0.3^*^	2.3 ± 0.5
VO_2peak_, mL·kg^−1^·min^−1^	15.4 ± 1.9	20 ± 1.4^*^	25.8 ± 3.5^*†^	38.4 ± 7.8
VO_2peak_, mL·kg FFM^−1^·min^−1^	32.5 ± 6.4	39.0 ± 5.3^*^	43.9 ± 7.1^*†^	−
Weight, kg	110.2 ± 21.4	99.4 ± 20.9	82.9 ± 8.0^*†^	59.7 ± 4.3
BMI, kg m^−2^	40.8 ± 7.7	35.8 ± 6.4^*^	30.8 ± 2.9^*†^	22.8 ± 1.4
Total FFM, kg	52.9 ± 9.1	51.5 ± 9.2	47.9 ± 4.7^*^	−
Leg FFM, kg	16.8 ± 3.1	16.5 ± 2.9	16.2 ± 1.9	−
Body fat %	50.0 ± 4.8	47.2 ± 4.8^*^	42.0 ± 5.2^*†^	−
Waist Circumference, cm	119.6 ± 16.7	113.8 ± 14.5	98.4 ± 9.4^*†^	−
SBP, mmHg	124.2 ± 15.5	117.8 ± 16.1	127.6 ± 14.9^†^	114.0 ± 9.9
DBP, mmHg	83.5 ± 8.3	76.5 ± 9.9^*^	80.5 ± 8.7	75.6 ± 7.5
Glucose, mg dL^−1^	98.0 ± 15.7	107.1 ± 30.4	99.8 ± 13.1	80.0 ± 10.7
Total cholesterol, mg dL^−1^	177.8 ± 44.5	202.8 ± 50.0^*^	188.3 ± 41.4	161.4 ± 16.3
HDL‐C, mg dL^−1^	50.5 ± 13.8	56.1 ± 12.8	61.4 ± 16.4^*^	56.9 ± 13.2
LDL‐C, mg dL^−1^	106.8 ± 35.7	123.7 ± 51.7	110.9 ± 38.7	89.9 ± 16.5
Triglycerides, mg dL^−1^	102.6 ± 55.9	114.7 ± 52.2	79.4 ± 35.4^†^	73.3 ± 31.6
*n* with MetS (%)	16 (51.6%)	8 (33.3%)	7 (38.9%)	−

Means ± SD. *P < 0.05 versus Very Low, ^†^ *P* < 0.05 versus Low.

### Circulating microRNAs

**Figure 1:**
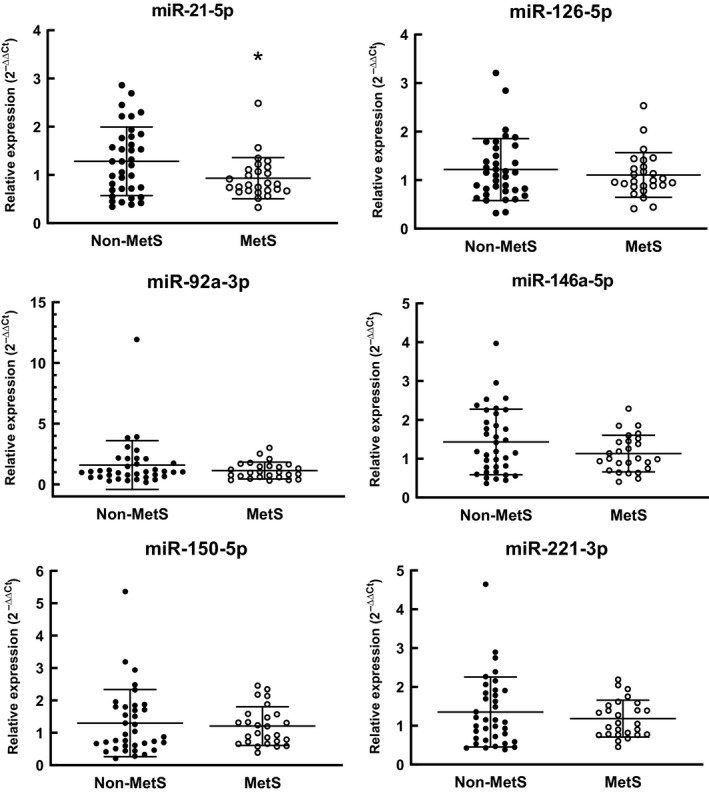
Relative expression (2^‐ΔΔCT^ method) of ci‐miR levels in serum of postmenopausal African American women with (MetS; *n* = 26) and without (Non‐MetS; *n* = 36) Metabolic Syndrome. Bars represent means ± SD. *P < 0.05 versus Non‐MetS.

Women with MetS had lower levels of ci‐miR‐21‐5p in comparison to women without MetS (0.93 ± 0.43 vs. 1.28 ± 0.71; *P* = 0.03) (Fig. 1). There were no significant differences between groups for any other ci‐miR. Next, subjects were separated based on presence/absence of each individual MetS risk factor. There were no significant differences in expression of any ci‐miR between groups separated based on any single risk factor.

**Figure 2:**
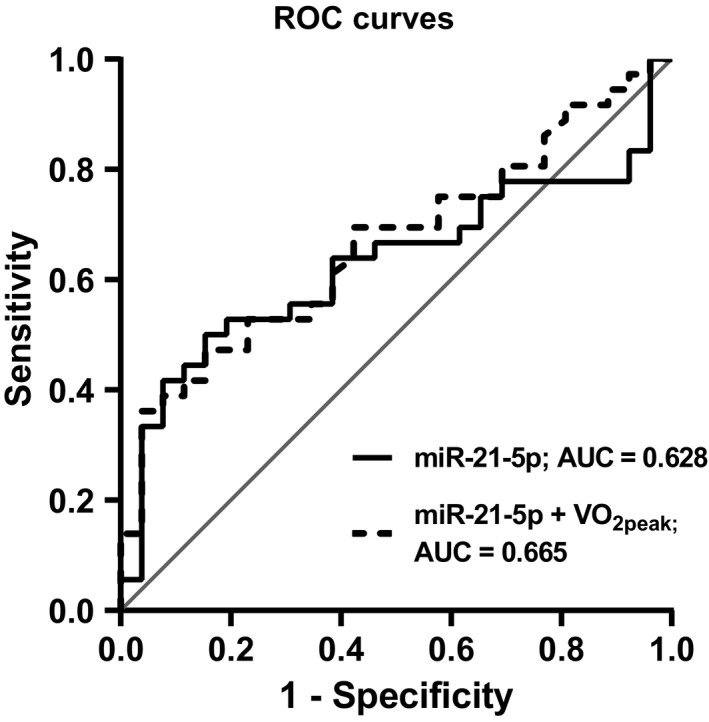
Receiver operating characteristic (ROC) curve analysis of the accuracy of ci‐miR‐21‐5p expression alone or in combination with VO_2peak_ (mL·kg^−1^·min^−1^) to differentiate women (*n* = 62) based on presence of MetS. Ci‐miR‐21‐5p: area under the curve (AUC) = 0.628, 95% CI = 0.488–0.769; *P* = 0.09. Ci‐miR‐21‐5p + VO_2peak_ (AUC = 0.665, 95% CI = 0.531–0.799; *P* = 0.03.

Since there were significant differences in ci‐miR‐21‐5p expression between women with and without MetS, we performed a Receiver‐Operating Characteristic (ROC) curve analysis to determine its ability to differentiate the two groups. The ROC curve revealed that ci‐miR‐21‐5p had poor utility in discriminating between the two groups (AUC = 0.628, 95% CI = 0.488–0.769, *P* = 0.09) (Fig. 2). Next, a logistic regression was used to determine whether the combination of ci‐miR‐21‐5p expression and VO_2peak_ increased the accuracy in discriminating between groups. Although now statistically significant, the combination had only slightly better accuracy (AUC = 0.665, 95% CI = 0.531–0.799, *P* = 0.03) than ci‐miR‐21‐5p alone.

**Figure 3:**
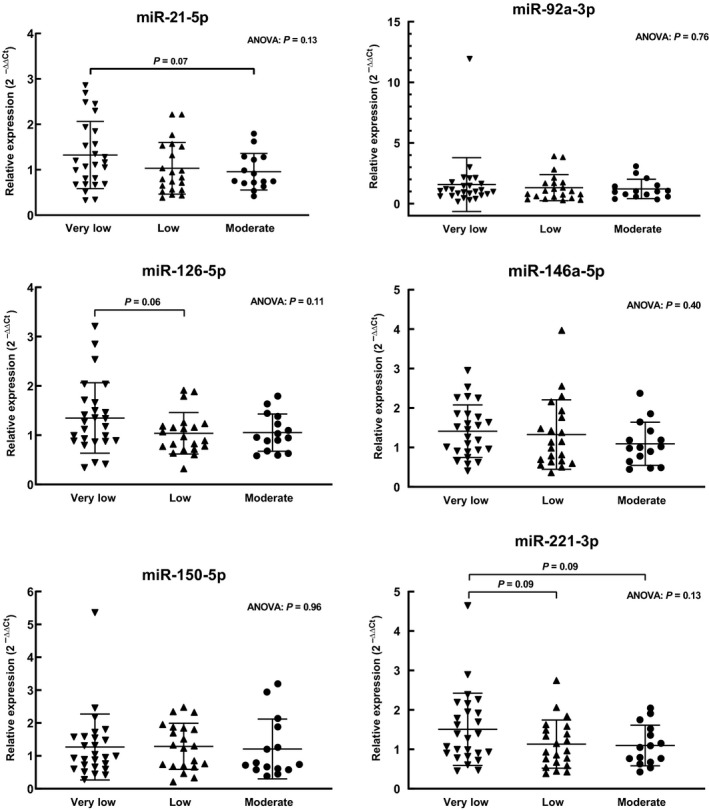
Relative expression (2^‐ΔΔCT^ method) of ci‐miR in serum of postmenopausal African American women grouped by cardiorespiratory fitness (CRF) level (Very Low [VO_2peak_ < 18.0 mL·kg^−1^ min^−1^; *n* = 26], Low [=18.0–22.0 mL·kg^−1^·min^−1^; *n* = 21], and Moderate [>22.0 mL·kg^−1^·min^−1^; *n* = 15]). Bars represent means ± SD.

**Figure 4:**
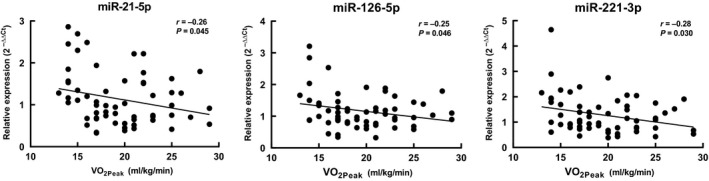
Correlations of ci‐miR‐21‐5p, ci‐miR‐126‐5p, and ci‐miR‐221‐3p with relative VO_2peak_ (mL·kg^−1^·min^−1^) *n* = 62.

One‐way ANOVAs comparing CRF groups were not significant for any ci‐miR. Pre‐planned post hoc comparisons revealed borderline significant differences for the endothelial function‐related ci‐miRs‐21‐5p, 126‐5p, and 221‐3p (Fig. 3). The Very Low CRF group tended toward higher levels of miR‐21‐5p in comparison to the Moderate CRF group (*P* = 0.07), higher miR‐126‐5p than the Low group (*P* = 0.06), and higher levels of miR‐221‐3p than the Low (*P* = 0.09) and Moderate (*P* = 0.09) groups. These miRs showed significant inverse correlations with relative VO_2peak_, ranging from *r* = −0.25 to −0.28 (*P* < 0.05) (Fig. 4).

### Endothelial migration rate

**Figure 5:**
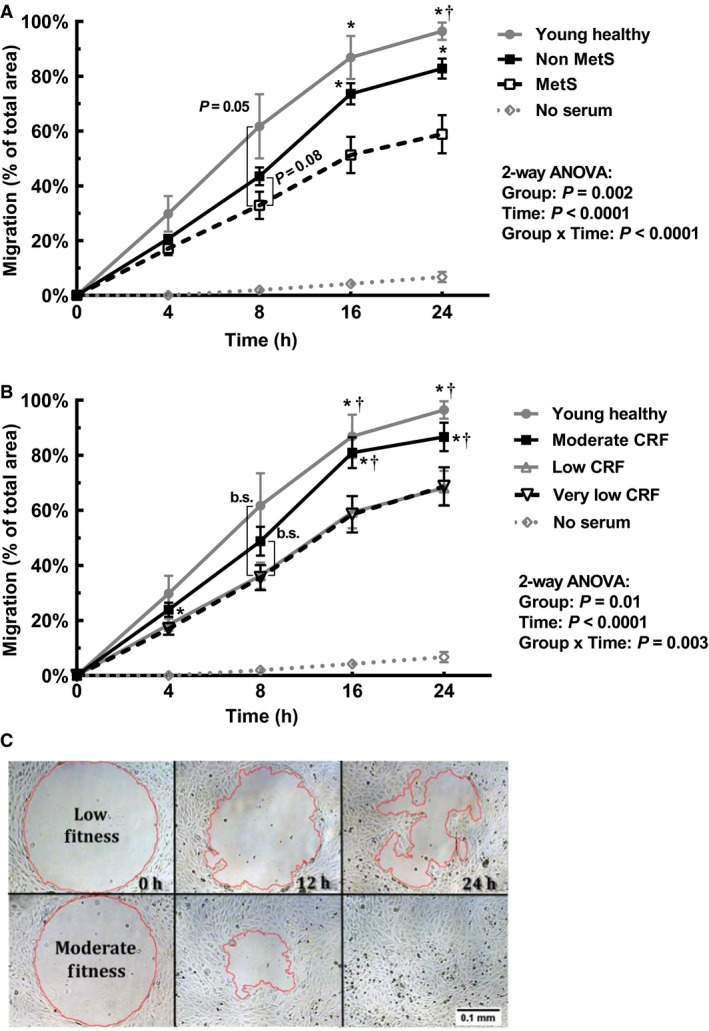
Endothelial migration in response to serum of postmenopausal women (A) grouped based on presence (MetS; *n* = 25)/absence (Non‐MetS; *n* = 37) of Metabolic Syndrome compared to healthy, young women (*n* = 7). *P < 0.05 versus MetS, †*P* < 0.05 versus Non‐MetS. (B) Endothelial migration in response to serum of women grouped by cardiorespiratory fitness (CRF) level (Very Low [VO_2peak_ < 18.0 mL·kg^−1^ min^−1^; *n* = 25], Low [=18.0–22.0 mL·kg^−1^·min^−1^; *n* = 21], and Moderate [>22.0 mL·kg^−1^·min^−1^; *n* = 16]) compared to young, healthy women (*n* = 7). *P < 0.05 versus Very Low, †*P* < 0.05 versus Low. There were borderline significant (b.s.) differences at 8 h for young, healthy versus Very Low (*P* = 0.071) and Low (*P* = 0.078), as well as Moderate versus Very Low (*P* = 0.065) and Low (*P* = 0.085). Means ± SD. (C) Representative images of endothelial migration in response to serum of postmenopausal women with Low and Moderate cardiorespiratory fitness (CRF). Endothelial cells were exposed to 5% serum in endothelial growth medium, migration was tracked over 24 h and calculated as (100 – (size of wound area at time point/size of initial wound area ×100)). The 0 h images were taken immediately upon addition of serum, while the 24 h image in response to serum of a woman with Moderate CRF shows 100% migration. Images were taken under a light microscope at 5× magnification.

Separating women based on presence/absence of MetS showed significant interaction (*P* < 0.001), group (*P* = 0.002), and time (*P* < 0.001) effects on endothelial migration rate as an index of endothelial repair capacity (Fig. 5A). At 8 h there were borderline significant differences, with the MetS group tending to be lower than the Non‐MetS (*P* = 0.08) and young, healthy group (*P* = 0.05). Migration in response to serum from women with MetS was significantly lower (*P* < 0.01) than those without MetS and the young, healthy group at 16 and 24 h. The young, healthy group induced greater migration than the Non‐MetS group at 24 h (*P* = 0.009). One‐way ANOVA analysis of AUC was significant (*P* = 0.002), revealing that endothelial migration AUC for the Non‐MetS group (1264 ± 412 migration∙hours) was significantly greater than the MetS group (912 ± 578 migration∙hours) (*P* = 0.006). The AUC for the young, healthy group (1570 ± 436 migration∙hours) was also greater than that of the MetS group (*P* = 0.002), but not significantly different from the Non‐MetS group (*P* = 0.13). No significant differences were observed when comparisons were made grouping subjects based on the presence of any single MetS risk factor.

Significant and borderline significant correlations with endothelial migration were observed for serum LDL concentration at 4 (*r* = −0.24) and 8 h (*r* = −0.24) (both *P* = 0.06), as well as triglyceride levels at 16 (*r* = −0.25; *P* = 0.048) and 24 h (*r* = −0.24; *P* = 0.06). There were similar borderline significant correlations between percent body fat and migration at 4 (*r* = −0.25; *P* = 0.06) and 8 h (*r* = −0.23; *P* = 0.07). Ci‐miR expression levels did not correlate with migration at any time point.

Comparison of migration by CRF groups using repeated measures ANOVA revealed significant interaction (*P* = 0.003), group (*P* = 0.01), and time (*P* < 0.001) effects (Fig. 5B and 5). At 4 h, serum from the Moderate group induced significantly greater migration than the Very Low group (*P* = 0.04). At 8 h, there were borderline significant differences with the young, healthy group being greater than the Very Low (*P* = 0.07) and Low (*P* = 0.08) groups, as well as the Moderate group being greater than the Very Low (*P* = 0.07) and Low (*P* = 0.09) groups. These differences were all significant at the 16‐ and 24‐h time points (*P* < 0.05). The young, healthy group was not significantly different from the Moderate group at any time point. One‐way ANOVA analysis of AUC was significant (*P* = 0.01), showing that AUC for the moderate group (1383 ± 404 migration∙hours) was not significantly different from the young healthy group, both of which were greater (*P* < 0.05) than the Low (1038 ± 479 migration∙hours) and Very Low (1025 ± 557 migration∙hours) groups.

## Discussion

The novel findings of our study are that in overweight/obese, postmenopausal African American women, (1) ci‐miR‐21‐5p is lower in those with MetS, (2) ci‐miRs related to endothelial function correlate with CRF, and (3) circulating blood‐borne factors are altered in association with CRF and MetS such that endothelial migration rate is lower in response to serum from women with lower CRF and with MetS. It is known that MetS predisposes individuals to CVD development, and our results provide additional evidence that this may occur partially through effects of altered blood‐borne factors on endothelial cell function. This may also be a mechanism by which lower CRF leads to CVD, while a relatively higher CRF is protective. Alterations in circulating factors may, therefore, have implications for CVD development in this high risk population.

Specific ci‐miRs are altered in association with cardiometabolic risk factors (Jansen et al. 2013; McManus et al. 2017), and are dysregulated with MetS (Njock and Fish, 2017). Of the ci‐miRs we investigated, only ci‐miR‐21‐5p was significantly different in women with MetS. Our results are in agreement with a study of Chinese women that likewise found ci‐miR‐21 to be lower in patients with MetS (He et al. 2016). In order to determine the ability of ci‐miR‐21‐5p to differentiate women with MetS, we performed an ROC curve analysis. The AUC revealed a poor accuracy in differentiating women with MetS, suggesting ci‐miR‐21‐5p is not a potential biomarker of MetS in this population. A previous study found enhanced accuracy in the diagnosis of coronary artery disease when the expression levels of ci‐miRs were combined with VO_2max_ test performance measures (Mayr et al. 2018). However, in our study the addition of VO_2peak_ to ci‐miR‐21‐5p expression only slightly increased the diagnostic ability, which remained poor.

The current literature has documented alterations in ci‐miRs based on acute exercise, training, and CRF level (Sapp et al. 2017; Sapp and Hagberg, 2019). Among the best characterized miRs are those enriched in the endothelium. Here, we observed correlations between CRF and the endothelial function‐related ci‐miRs‐21‐5p, 126‐5p, and 221‐3p, despite a low range of VO_2peak_ values (all subjects <30 mL·kg^−1^·min^−1^). A similar correlation of ci‐miR‐21 with VO_2max_ (*r* = −0.20) was previously found in a healthy cohort of middle‐aged men and women (Bye et al. 2013). In a study of Chinese individuals, serum ci‐miR‐126 levels were higher in those with MetS compared to healthy controls, and ci‐miR‐126 was lower in the most physically active MetS patients when split into quartiles (Zhou et al. 2014). While all subjects in our study were sedentary, we observed a similar negative correlation with ci‐miR‐126‐5p levels and CRF in our African American cohort, although there were no differences due to MetS.

Another factor that may have influenced ci‐miRs in our cohort was body fat. When separated by CRF, percent body fat differed significantly between groups. Still, CRF groups differed by only 3–8% body fat on average and all groups were classified as obese with a BMI greater than 30 kg m^−^^2^. Additionally, all but five participants had a waist circumference greater than 88 cm (risk criteria for MetS). In a study of lean and obese individuals with and without diabetes, Nunez Lopez et al. (2017) found positive correlations between percent body fat and both ci‐miR‐126 and ci‐miR‐146a, although we did not observe significant correlations with any ci‐miRs. Thus, a future study in a similar cohort to ours including a greater range of body fat levels may be needed to explore the relationship between body fat and cardiovascular ci‐miRs.

Importantly, several ci‐miRs incorporated by the endothelium play mechanistic roles in endothelial repair processes (Zhang et al. 2010; Jansen et al. 2013; Njock and Fish, 2017) and the pathogenesis of vascular diseases (Zernecke et al. 2009; Bronze‐da‐Rocha, 2014; Dlouhá and Hubáček, 2017). Metabolic risk factors are known to have injurious effects on the endothelium directly through the circulation, and MetS is associated with endothelial cell dysfunction, increased endothelial injury, and reduced ability for repair (Jialal et al. 2010a). Interestingly, miR‐21 has been shown to stimulate endothelial cell migration (Luo et al. 2017), likely via its actions on a number of targets. In order to determine the potential impacts of MetS and CRF on endothelial repair capacity via circulating factors, we tested the effects of subjects’ serum on endothelial cell migration. We found that serum from women with Moderate CRF and without MetS induced a greater migration response of endothelial cells as compared to serum from their counterparts who were less fit and had MetS. However, we found no association between the expression of ci‐miR‐21‐5p (or any other ci‐miR) and endothelial migration rate, suggesting that other factors in serum were responsible for the altered migration rate.

Previous studies suggest an impaired capacity for endothelial wound repair in individuals with MetS that is mediated, at least in part, by physical inactivity (Jialal et al. 2010a; Jialal et al. 2010b; Sonnenschein et al. 2011). The impact of CRF level on the ability of endogenous endothelial cells to repair has been less well studied. We found that the circulating factors in serum from women with a Moderate CRF level resulted in a favorably higher endothelial migration rate compared to those with lower CRF levels. Thus, a moderate level of CRF may protect the endothelium by maintaining circulating factors that promote endothelial repair. The fact that endothelial migration in response to serum from those postmenopausal women with Moderate CRF was on average not different from young, healthy women supports this idea.

Our results further support an inhibitory effect of circulating factors in women with MetS on the migratory ability of endothelial cells. This effect on endothelial migration may be mediated by a number of factors. The only factors to display borderline significant correlations with migration in our study were LDL, triglycerides, and body fat percentage. Elevated circulating lipids and cholesterol are known to promote endothelial dysfunction and injury (Pirro et al. 2008; Kim et al. 2012). However, given the weak correlations (*r* = 0.24–0.25), these factors are not likely to fully explain the observed differences in endothelial migration in our study. Other circulating factors previously shown to be unfavorably altered in individuals with MetS include plasma insulin, endothelin‐1, plasminogen activator inhibitor‐1, nitric oxide, C‐reactive protein, and interleukin‐6 (Briançon‐Marjollet et al. 2014; Chedraui et al. 2014; Ahirwar et al. 2015). Future studies should seek to determine the specific circulating factors potentially contributing to impaired endothelial migration with MetS.

In conclusion, ci‐miRs related to endothelial function and cardiovascular health are associated with CRF in overweight/obese, postmenopausal, African American women. Women with MetS had lower ci‐miR‐21‐5p and their serum had reduced capacity to induce endothelial migration. Serum from moderately fit women caused greater migration than those with lower CRF levels. Therefore, in this high risk group for CVD, maintaining a healthy metabolic profile and at least a Moderate CRF may be protective for cardiovascular health through maintenance of circulating factors that promote endothelial repair. The importance of CRF is further increased considering a Moderate CRF level decreases the risk of MetS and related comorbidities (Kaminsky et al. 2013; Adams‐Campbell et al. 2016). Habitual exercise is a useful lifestyle intervention to both increase CRF and decrease MetS risk factors, although beneficial effects of exercise interventions on cardiovascular health in postmenopausal women are not always apparent, related to loss of estrogen (Moreau and Ozemek, 2017). Thus, future studies are warranted to uncover mechanisms underlying cardiovascular adaptions and optimal training interventions for postmenopausal women.

## Conflict of Interest

No conflicts of interest, financial or otherwise, are declared by the authors.
